# Quantitative assessment of exposure to fecal contamination in urban environment across nine cities in low-income and lower-middle-income countries and a city in the United States

**DOI:** 10.1016/j.scitotenv.2021.151273

**Published:** 2022-02-01

**Authors:** Yuke Wang, Wolfgang Mairinger, Suraja J. Raj, Habib Yakubu, Casey Siesel, Jamie Green, Sarah Durry, George Joseph, Mahbubur Rahman, Nuhu Amin, Md. Zahidul Hassan, James Wicken, Dany Dourng, Eugene Larbi, Lady Asantewa B. Adomako, Ato Kwamena Senayah, Benjamin Doe, Richard Buamah, Joshua Nii Noye Tetteh-Nortey, Gagandeep Kang, Arun Karthikeyan, Sheela Roy, Joe Brown, Bacelar Muneme, Seydina O. Sene, Benedict Tuffuor, Richard K. Mugambe, Najib Lukooya Bateganya, Trevor Surridge, Grace Mwanza Ndashe, Kunda Ndashe, Radu Ban, Alyse Schrecongost, Christine L. Moe

**Affiliations:** a Center for Global Safe Water, Sanitation, and Hygiene, Hubert Department of Global Health, Rollins School of Public Health, Emory University, Atlanta, GA, USA; b Water Global Practice, The World Bank, Washington, DC, USA; c Environmental Interventions Unit, Infectious Disease Division, International Centre for Diarrhoeal Disease Research, Bangladesh (icddr,b), Dhaka, Bangladesh; d Data Analysis and Technical Assistance Limited, Dhaka, Bangladesh; e WaterAid, Phnom Penh, Cambodia; f Training Research and Networking for Development (TREND), Accra, Ghana; g Council for Scientific and Industrial Research Water Research Institute, Accra, Ghana; h Department of Civil Engineering, Kwame Nkrumah University of Science and Technology, Kumasi, Ghana; i Development Planning Unit, Kumasi Metropolitan Assembly, Kumasi, Ghana; j Wellcome Research Laboratory, Christian Medical College, Vellore, India; k School of Civil and Environmental Engineering, Georgia Institute of Technology, Atlanta, GA, USA; l Water Supply and Mapping, WE Consult, Maputo, Mozambique; m Initiative Prospective Agricole et Rurale (IPAR), Dakar, Senegal; n Department of Disease Control and Environmental Health, Makerere University School of Public Health, Kampala, Uganda; o Department of Environment and Public Health, Kampala Capital City Authority, Kampala, Uganda; p Deutsche Gesellschaft für Internationale Zusammenarbeit (GIZ) GmbH, Lusaka, Zambia; q Department of Public Health, Lusaka City Council, Lusaka, Zambia; r Department of Environmental Health, Faculty of Health Science, Lusaka Apex Medical University, Lusaka, Zambia; s Bill & Melinda Gates Foundation, Seattle, WA, USA

**Keywords:** Exposure assessment, Multi-city, WASH, Fecal, Pathway, LLMIC

## Abstract

**Background:**

During 2014 to 2019, the SaniPath Exposure Assessment Tool, a standardized set of methods to evaluate risk of exposure to fecal contamination in the urban environment through multiple exposure pathways, was deployed in 45 neighborhoods in ten cities, including Accra and Kumasi, Ghana; Vellore, India; Maputo, Mozambique; Siem Reap, Cambodia; Atlanta, United States; Dhaka, Bangladesh; Lusaka, Zambia; Kampala, Uganda; Dakar, Senegal.

**Objective:**

Assess and compare risk of exposure to fecal contamination via multiple pathways in ten cities.

**Methods:**

In total, 4053 environmental samples, 4586 household surveys, 128 community surveys, and 124 school surveys were collected. *E. coli* concentrations were measured in environmental samples as an indicator of fecal contamination magnitude. Bayesian methods were used to estimate the distributions of fecal contamination concentration and contact frequency. Exposure to fecal contamination was estimated by the Monte Carlo method. The contamination levels of ten environmental compartments, frequency of contact with those compartments for adults and children, and estimated exposure to fecal contamination through any of the surveyed environmental pathways were compared across cities and neighborhoods.

**Results:**

Distribution of fecal contamination in the environment and human contact behavior varied by city. Universally, food pathways were the most common dominant route of exposure to fecal contamination across cities in low-income and lower-middle-income countries. Risks of fecal exposure via water pathways, such as open drains, flood water, and municipal drinking water, were site-specific and often limited to smaller geographic areas (i.e., neighborhoods) instead of larger areas (i.e., cities).

**Conclusions:**

Knowledge of the relative contribution to fecal exposure from multiple pathways, and the environmental contamination level and frequency of contact for those “dominant pathways” could provide guidance for Water, Sanitation, and Hygiene (WASH) programming and investments and enable local governments and municipalities to improve intervention strategies to reduce the risk of exposure to fecal contamination.

## Highlights

• Standardized multi-pathway fecal exposure assessments were conducted in ten cities.
• Thousands of environmental samples and behavior surveys were collected.
• Food pathways were the most dominant exposure pathway across cities in LLMICs.
• Dominant fecal exposure pathways varied by neighborhood within cities.
• Results from multi-pathway fecal exposure assessment can guide WASH programming.

## Graphical abstract

**Figure:**
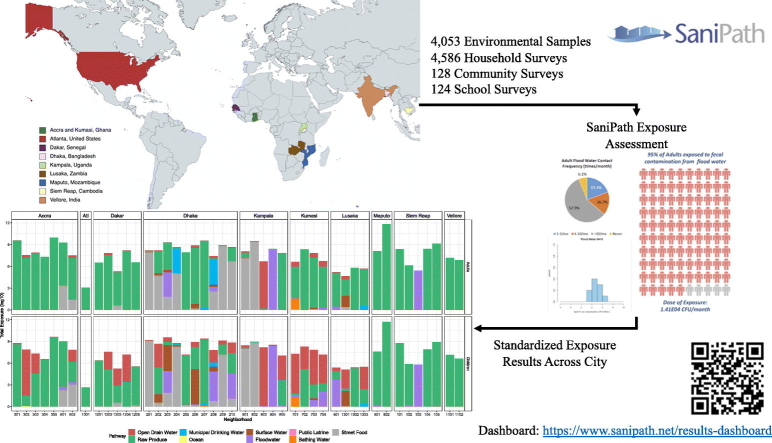

## 1 Introduction

More than half of the world's population (55% in 2018) live in urban areas. In low-income and lower-middle-income countries (LLMICs), rapid urbanization is predicted for 2018–2050, which result in a projected 68% of world's population residing in urban areas by 2050 (United Nations, 2018). Globally, the urban population increased dramatically from 751 million in 1950 to 4.2 billion in 2018 and a quarter of the urban population lives in informal settings especially slums (United Nations, 2018; United Nations Human Settlements Programme, 2016). In slums, the average cost of owning a household toilet is not affordable for most inhabitants; in some instances, there may be inadequate space for the tenants residing in the predominantly shack structures for construction of toilet facilities (Peprah et al., 2015). Consequently, the majority of the tenants patronize public latrines or resort to open defecation. In LLMICs, rapid urbanization and population growth have far outpaced the development of urban infrastructure creating huge gaps in access to safe water and sanitation.

Inadequate sanitation and poor fecal sludge management contribute to the spread of fecal contamination and associated pathogens in the environment (Robb et al., 2017), and exposure to fecal contamination poses the risk of developing gastroenteritis, environmental enteric dysfunction, and stunting (Ngure et al., 2014). Each year there are an estimated 1.7 billion cases of pediatric diarrhea and 525,000 deaths attributed to diarrheal disease among children under five across the globe (World Health Organization, 2015). Fecal matter in the environment may be ingested by humans through multiple environmental pathways. The relative importance of various pathways of exposure to fecal contamination in the environment, and transmission routes for enteric pathogens, are likely site-specific (Robb et al., 2017; Wang et al., 2017; Julian, 2016). Therefore, the effect of an intervention to reduce this exposure and associated disease outcomes may vary by location. Fecal exposure assessment studies have been conducted in different countries focusing on specific pathways, as determined by site-specific information or expert opinion (Antwi-Agyei et al., 2015; Gretsch et al., 2016; Owusu-Ansah et al., 2017). However, there was no standardized procedure for conducting fecal exposure assessment for multiple pathways. SaniPath Exposure Assessment (Robb et al., 2017; Wang et al., 2017; Raj et al., 2020; SaniPath Research Team, 2015) provides a systematic, standard procedure and has been used to evaluate the contributions of multiple pathways to the total exposure to fecal contamination in the environment and guide evidence-based decision making about water, sanitation, and hygiene (WASH) interventions targeted to “dominant pathways”.

The current study presents findings for multi-pathway exposure assessments from ten cities: Accra, Ghana; Vellore, India; Maputo, Mozambique; Siem Reap, Cambodia; Atlanta, United States; Dhaka, Bangladesh; Kumasi, Ghana; Lusaka, Zambia; Kampala, Uganda; Dakar, Senegal.

## 2 Methods

**Fig. 1:**
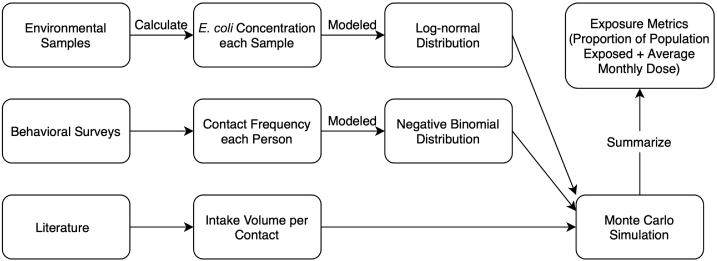
Conceptual diagram of the SaniPath exposure assessment.

The SaniPath exposure assessment (Robb et al., 2017; Wang et al., 2017, Wang et al., 2018) aims at quantifying fecal contamination ingested by populations (adults or children) living in urban environments through different exposure pathways in standardized metrics and compares the relative importance of these exposure pathways to inform evidence-based decision making about WASH policies and interventions. In this study, we defined exposure pathways as links from environmental compartments (i.e., reservoirs) to ingestion. Fig. 1 shows the conceptual diagram of the SaniPath exposure assessment. Environmental samples were collected and tested for fecal indicator bacteria (*E. coli*) to estimate levels of fecal contamination in different environmental compartments. Meanwhile, behavioral information was collected by surveys to estimate the number of contacts with those contaminated environmental compartments. Along with intake volume per contact from literatures (Raj et al., 2020), standardized metrics of exposure, including the proportion of population exposed to the fecal contamination and the average exposure dose (i.e., the amount of fecal contamination ingested) per month, were estimated and compared by pathway. The results of the exposure assessment could help identify the dominant pathway(s) of exposure to fecal contamination within a neighborhood and guide evidence-based decision making to prioritize and appropriately target WASH interventions.

In 2013, the SaniPath team at the Center of Global Safe Water, Sanitation, and Hygiene (CGSW) at Emory University developed the SaniPath Exposure Assessment Tool (Raj et al., 2020; SaniPath Research Team, 2015), hereafter referred to as the SaniPath Tool. The SaniPath Tool guides the users to conduct a systematic, standardized SaniPath exposure assessment that includes key informant interviews, transect walks, behavioral surveys, collection of relevant environmental samples, and microbiological analyses (Raj et al., 2020). Data was collected by mobile devices using the KoBoToolbox platform (The Harvard Humanitarian Initiative, 2014) and analyzed in real time using Amazon Web Services (EC2 instances). The results are visualized on an online dashboard and summarized in an automated report.

### 2.1 Study sites

During 2014 to 2019, the SaniPath Tool was deployed in 45 neighborhoods in ten cities from nine countries. Each deployment was a collaboration between CGSW at Emory University and in-country collaborators (Table 1). Local partners included local research institutes, non-governmental organizations (NGOs), consulting companies, or government agencies with the capacity to conduct surveys and the required laboratory testing. Multiple stakeholder meetings were held to describe the goals of the assessment and understand the information needs of the stakeholders, assess the city infrastructure, identify potential exposure pathways, discuss candidate study neighborhoods and criteria for choosing study neighborhoods, and support dissemination of the SaniPath assessment results. In each country, Institutional Review Board (IRB) approval, permissions from city authorities, written informed consent from adult participants, and assent for school children were obtained before conducting the SaniPath exposure assessment.

**Table 1:** SaniPath exposure assessment sites by city.

Country	Time	City	Neighborhoods	Partners
Bangladesh	04/2017–07/2017	Dhaka	Badda, Dhanmondi, Gabtoli Bus Terminal, Gendaria Railway Station, Gulshan, Hazaribagh, Kalshi Mirpure, Kamalapur Ticket Counter, Motijhil, Uttar Khan	World Bank; Data Analysis and Technical Assistance (DATA); International Centre for Diarrhoeal Disease Research, Bangladesh (icddr, b)
Cambodia	09/2016–11/2016	Siem Reap	Chong Kaosou, Kumruthemey (formal), Kumruthemey (informal), Steung Thumey, Veal/Trapangses	Cambodian Ministry of Public Works and Transport; The Community and Engagement and Development Team (CEDT); Water for Cambodia; WaterAid Cambodia
Ghana	03/2016–07/2016	Accra	Adabraka, Chorkor, Kokomlemle, Ringway, Shiabu	Accra Metropolitan Assembly; Ministry of Sanitation and Water Resources; TREND; Water Research Institute (WRI)
	09/2018–10/2018	Accra	Mataheko, Osu Alata	
Ghana	08/2018–11/2018	Kumasi	Ahodwo, Dakodwom, Fante New Town, Moshie Zongo	Kumasi Metropolitan Assembly (KMA); Kwame Nkrumah University of Science and Technology (KNUST); Ministry of Sanitation and Water Resources; TREND; Water Research Institute (WRI)
India	02/2014–04/2014	Vellore	Chinna Allapuram, Old Town	Christian Medical College (CMC); University of Brighton
Mozambique	03/2015–05/2016	Maputo	Control, Intervention	Georgia Institute of Technology (GT); National Laboratory for Food and Water Hygiene, Mozambique; WE consult
Senegal	11/2019–01/2020	Dakar	Wakhinane Nimzatt, Medina Gounass, Djiddha Thiaroyye Kao (DTK), Rufisque Est, Sicap Liberte	Initiative Prospective Agricole et Rurale (IPAR); Institut Pasteur de Dakar (IDP)
Uganda	11/2018–12/2018	Kampala	Central, Kawempe, Makindye, Nakawa, Rubaga	Kampala Capital City Authority (KCCA); Makerere University School of Public Health
United States	10/2016–12/2016	Atlanta	Peoplestown	
Zambia	03/2018	Lusaka	Kanyama	GiZ (Deutsche Gesellschaft fuer Internationale Zusammenarbeit); Lusaka City Council (LCC); University of Zambia, Veterinary Medicine
	10/2019	Lusaka	Chawama, Chazanga, George	

All the deployments were driven by the demand to evaluate and compare exposure to fecal contamination through multiple pathways by geographic area (i.e., neighborhood) and population (i.e., age group). Low-income urban neighborhoods were prioritized to understand the “worst case scenarios” and risks to the vulnerable populations residing in these communities. When there were more resources or additional research interests (e.g., in Accra, Ghana and Dhaka, Bangladesh), neighborhoods were selected to include variation in socioeconomic status (SES), types of settlement, WASH conditions, and geographic locations within the city (Amin et al., 2019).

### 2.2 Data collection and sample testing

Nine exposure pathways (bathing water, flood water, municipal drinking water, surface water, ocean water, open drain, public or shared latrines, raw produce, street food) were detailed defined in the standardized version of the Tool (Raj et al., 2020). Where necessary, additional pathways (e.g., other drinking water sources such as shallow well water, well water, borehole water, bottled water, spring water, and ice) were included based on key informant interviews conducted before data collection. In total, 4053 environmental samples, 4586 household surveys, 128 community surveys, and 124 school surveys were collected from 45 neighborhoods in ten cities (Tables 2, S1).

**Table 2:** Numbers of environmental samples and behavior surveys collected by city.

Country	City	Year	Sample size					
			Neighborhoods	Pathway	Environmental samples	Household surveys	Community surveys (participants)	School surveys (participants)
Ghana	Accra	2016	5	7	688	821	22 (293)	12 (315)
Ghana	Accra	2018	2	9	149	200	8 (127)	8 (120)
United States	Atlanta	2016	1	4	47	23	N/A	N/A
Senegal	Dakar	2020	5	5	300	500	20 (300)	20 (300)
Bangladesh	Dhaka	2017	10	10	1000	823	28 (501)	35 (597)
Uganda	Kampala	2018	5	9	382	548	10 (112)	9 (114)
Ghana	Kumasi	2018	4	9	282	400	16 (240)	16 (320)
Zambia	Lusaka	2018	1	8	170	100	4 (79)	4 (73)
Zambia	Lusaka	2019	3	9	250	300	12 (219)	12 (240)
Mozambique	Maputo	2016	2	7	376	261	N/A	N/A
Cambodia	Siem Reap	2016	6	5	303	410	N/A	N/A
India	Vellore	2014	2	5	106	200	8 (117)	8 (151)
	Total		45	87	4053	4586	128 (1988)	124 (2230)

For each neighborhood, the minimum sample size requirement was ten environmental samples per pathway, 100 household surveys, four community surveys of 15–20 people, and four school surveys of 15–20 children at age 10–12 years old. Ten soil samples were also collected as a proxy for the overall fecal contamination of the environment. We did not include soil as an exposure pathway in this assessment due to lack of reliable information of soil ingestion, which could vary substantially by site. Additional samples and surveys were collected whenever the geographic area was large or there was heterogeneity in population or sanitation infrastructures within the neighborhood (e.g., mixed formal and informal settlements). When sampling locations were rare (e.g., municipal water taps) in the neighborhood, multiple samples from the same sampling location were collected on different days. Sample sites were usually selected based on the usage/popularity of the sites as determined from transect walks and key informant interviews. In Lusaka and Kampala, where the size of some neighborhoods was large, systematic grid sampling was used (SaniPath Research Team, 2018) to distribute sampling sites across the neighborhood. Most of the deployments were conducted during the peak diarrhea season/rainy season.

All the environmental samples were analyzed and quantified for *E. coli*, as a fecal indicator. Either membrane filtration with m-ColiBlue24® (Hach Company, Loveland, CO) broth media or Chromocult® Coliform Agar (EMD MilliporeSigma, Burlington, MA), or the IDEXX-Colilert-24® and the Quanti-Tray/2000 (IDEXX Laboratories, Westbrook, ME) was used. Multiple dilutions, increased by either 10-fold or 100-fold, were tested for each sample to accurately measure the concentration of *E. coli*, which may show large variation between samples, even for the same sample type. We calculated the concentration of *E. coli* for each sample by selecting and averaging concentrations from multiple dilutions. Replicate readings at multiple dilutions also allow validation of the results for quality control (Raj et al., 2020).

Household surveys were conducted with selected adults according to the following criteria: living in the sampled household, managing the water, and being familiar with the daily activities of the children. Households were randomly selected among eligible households (SaniPath Research Team, 2018) that had an adult matching the above criteria and at least one child between 5 and 12 years old. Community and school surveys were conducted with 15–20 participants in each survey. Separate community surveys for men and women were recommended at each site, and participants were recruited through community partners. Eligible participants were defined as adults living in the neighborhood of interest, with a preference for those having at least one child between 5 and 12 years old. Separate school surveys for boys and girls were also recommended when local partners identified potential bias in responses of respondents in instances when they were around members of the opposite gender. Schoolchildren were eligible for participation if they were between 10 and 12 years old and lived in the study neighborhood. Community and school surveys were conducted in group meeting settings and relied on voting methods to record responses (SaniPath Research Team, 2018). Community members voted anonymously using colored tokens or ballots, and schoolchildren covered their eyes and voted with tokens in raised hands to reduce the potential bias in group settings. Information about private toilets were also collected in household surveys and the results were presented in the Supplementary Material S2.

The rationale and details of study settings (e.g., sample size, sampling location, inclusive criteria etc.) for environmental sample collection, laboratory methods, and survey methods were described by Raj et al. (2020).

### 2.3 Data analysis

To quantify exposure to fecal contamination, information on both the contamination level of the environmental samples and the frequency of behaviors leading to contact with the environment were required. The concentrations of *E. coli* in the environmental samples, measured from replicate readings from multiple dilutions, were modeled using log-normal distributions. Frequencies of contact with each environmental compartment were measured in frequency ranges (e.g., 1–5 times per month) and were modeled using negative binomial distributions with censored data. The parameters of those distributions varied by varied by pathway, neighborhood, and population (for frequency of contact). These parameters were estimated using Bayesian frameworks by JAGS (Plummer, 2003) and 1000 iterations of Monte Carlo simulation (with estimated parameters) were conducted to assess the exposure to fecal contamination by pathway, neighborhood, and age group (Fig. 1). The details of the assumptions, parameters, and models are documented in Raj et al. (2020). Outcomes of quantitative exposure assessment (including the average dose of exposure to fecal contamination and the percent of population exposed to fecal contamination) were used to compare magnitude and patterns of exposure, by pathway, across cities. All data cleaning, manipulation, analysis, and visualization were done with R version 3.4.4 (R Core Team, 2013). All the data used in this analysis were published on Dataverse (SaniPath Research Team, 2021).

## 3 Results

### 3.1 Environmental contamination

**Fig. 2:**
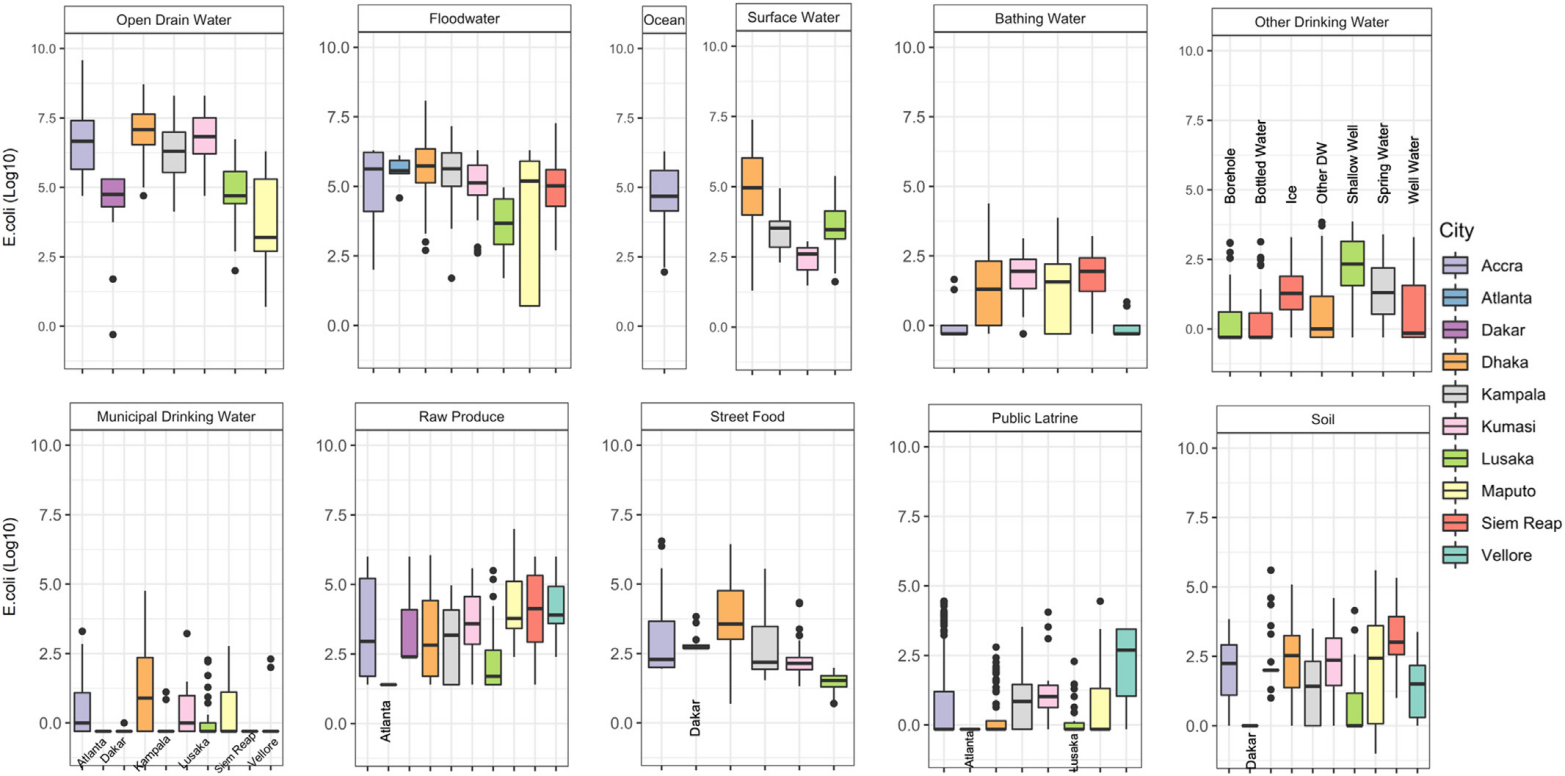
Environmental fecal contamination across cities for different sample types. The box of boxplot presents 25th percentile (Q1), median, and 75th percentile (Q3). The whiskers represent the Q1–1.5IQR (interquartile range) and Q3 + 1.5IQR. The unit of *E. coli* concentration for all the water samples is either colony-forming unit (CFU) for membrane filtration or most probable number (MPN) for IDEXX per 100 mL. The *E. coli* units of concentration are CFU or MPN per serving for produce and street food, CFU or MPN per swab for public latrine swabs, and CFU or MPN per gram of soil. The results are color coded by city. Labels at the bottom indicate boxes with hidden colors. Types of Other Drinking Water samples are labeled on the top of the boxes. (For interpretation of the references to color in this figure legend, the reader is referred to the web version of this article.)

Fig. 2 shows the levels of fecal contamination (*E. coli* concentration) across cities by sample type; corresponding descriptive statistics can be found in Table 3. Ordered from high to low fecal contamination levels, water samples were: open drain water, flood water, surface water and ocean water, bathing water, other drinking water, and municipal drinking water. However, we observed large variation between cities in the fecal contamination levels of specific sample types, sometimes changing the order of contamination levels. For example, in Dhaka the municipal drinking water was significantly more contaminated compared to other sources of drinking water (*p* = 0.008). Among the study cities, Dhaka had the highest fecal contamination levels in municipal drinking water with a mean *E. coli* concentration of 3.36 log10 *E. coli* MPN/100 mL, compared to other cities with mean concentration less than 1.86 log10 CFU or MPN/100 mL. Samples of shallow well water (mean 2.71 log10 *E. coli* CFU/100 mL) in Lusaka and ice (mean 2.23 log10 *E. coli* CFU/100 mL) in Siem Reap also had high levels of fecal contamination. Lusaka had low levels of fecal contamination in samples of flood water and open drain water, and Dakar also had a low level of fecal contamination in samples of open drain water. Raw produce was often fecal contaminated, with mean concentrations of *E. coli* ranging from 1.40 log10 MPN/serving in Atlanta to as high as 6.36 log10 CFU/serving in Maputo. Street food was most contaminated in Accra with a mean concentration of 5.52 log10 *E. coli* CFU/serving. A low percentage of street food in other cities was also highly contaminated. Public latrine swabs had relatively low contamination levels across all cities except in Vellore, where the mean concentration was 3.29 log10 *E. coli* MPN/swab. Soil samples showed variation both within, and between, cities. Dakar and Maputo had the highest concentrations of *E. coli* in soil, while Kampala and Lusaka had the lowest. No correlation was found between the fecal contamination levels in soil and the fecal contamination level of any other sample type.

**Table 3:** The mean and range of levels of fecal contamination (*E. coli*) in log10 scale across cities by sample type.

City	Lab method	Drain water	Flood water	Ocean water	Surface water	Bathing water	Other drinking water	Municipal drinking water	Raw produce	Street food	Public latrine	Soil
Accra	MF	7.99 (4.70–9.58)	5.89 (2.00–6.30)	5.59 (1.95–6.30)	N/A	0.88 (–0.30–1.65)	N/A	1.86 (−0.30–3.30)	5.24 (1.40–6.00)	5.52 (1.95–6.55)	3.29 (−0.15–4.45)	2.98 (0–3.85)
Atlanta	IDEXX	N/A	5.76 (4.58–6.11)	N/A	N/A	N/A	N/A	−0.30 (−0.30−−0.30)	1.40 (1.40–1.40)	N/A	0.17 (−0.15–0.92)	3.40 (0–4.21)
Dakar	MF	5.00 (−0.30–5.30)	N/A	N/A	N/A	N/A	N/A	-0.30 (−0.30–0)	4.79 (2.40–6.00)	2.88 (2.67–3.83)	N/A	4.84 (1–5.60)
Dhaka	IDEXX	7.57 (4.70–8.71)	6.54 (2.70–8.08)	N/A	6.08 (1.30–7.38)	3.06 (−0.30–4.38)	2.21 (−0.30–3.84)	3.36 (−0.30–4.76)	4.83 (1.40–6.06)	5.11 (0.69–6.44)	1.26 (−0.15–2.80)	3.76 (0–5.09)
Kampala	MF	7.15 (4.13–8.30)	6.14 (1.70–7.16)	N/A	4.12 (2.30–4.94)	N/A	2.34 (−0.30–3.40)	−0.01 (−0.30–1.11)	4.14 (1.40–4.97)	4.37 (1.54–5.56)	2.31 (−0.15–3.53)	2.42 (0–3.51)
Kumasi	MF	7.46 (4.70–8.30)	5.64 (2.60–6.30)	N/A	2.71 (1.48–3.05)	2.38 (−0.30–3.13)	N/A	1.71 (−0.30–3.22)	4.45 (1.40–5.59)	3.12 (1.32–4.34)	2.80 (−0.15–4.05)	3.39 (0–4.60)
Lusaka	IDEXX	5.56 (2.00–6.74)	4.38 (1.70–4.97)	N/A	4.48 (1.61–5.38)	N/A	2.71 (−0.30–3.86)	1.05 (−0.30–2.27)	4.03 (1.40–5.50)	1.56 (0.70–1.99)	0.81 (−0.15–2.28)	2.60 (0–4.15)
Maputo	MF	5.27 (0.70–6.30)	5.76 (0.70–6.30)	N/A	N/A	2.63 (−0.30–3.88)	N/A	1.46 (−0.30–2.77)	6.36 (2.40–7.00)	N/A	2.78 (−0.15–4.45)	4.28 (−1.00–5.60)
Siem Reap	MF	N/A	5.83 (2.70–7.27)	N/A	N/A	2.50 (−0.30–3.21)	2.11 (−0.30–3.30)	−0.30 (−0.30−0.30)	5.14 (1.40–6.00)	N/A	N/A	4.10 (1.00–5.33)
Vellore	MF	N/A	N/A	N/A	N/A	0.18 (−0.30–0.85)	N/A	1.23 (−0.30–2.30)	5.23 (2.40–6.00)	N/A	3.04 (−0.15–3.45)	2.32 (0–3.38)

Types of “Other drinking water” varied by city and included shallow well water, well water, borehole water, bottled water, spring water, and ice. The unit of *E. coli* concentration for all the water samples was either colony-forming unit (CFU) for membrane filtration (MF) or most probable number (MPN) for IDEXX per 100 mL. *E. coli* units of concentration are CFU or MPN per serving for produce and street food, CFU or MPN per swab for public latrine swabs, and CFU or MPN per gram of soil. All concentrations are shown in log10 scale. N/A indicates that no samples of that type were collected because the exposure pathway was not included in the city.

### 3.2 Behavior frequency

**Fig. 3:**
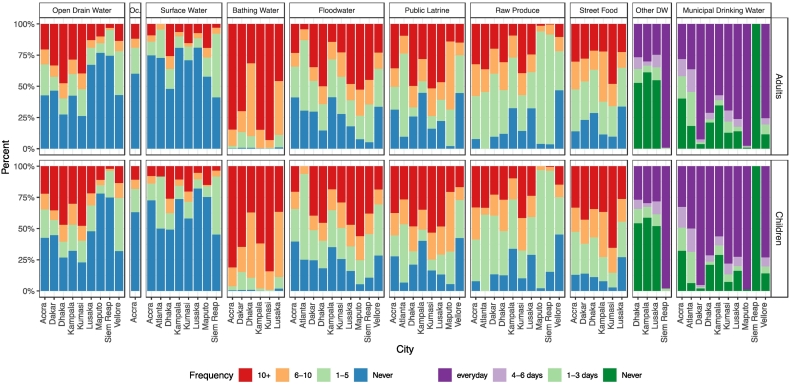
Combined behavior frequency of contacting various environmental pathways across cities from household surveys, community surveys, school surveys. The frequency categories vary by pathway. For open drains, ocean water (Oc.), and surface water, the categories are never, 1 to 5 times per month, 6 to 10 times per month, and more than 10 times per month. For bathing water, flood water, public latrines, raw produce, and street food, the categories are never, 1 to 5 times per week, 6 to 10 times per week, and more than 10 times per week. For municipal drinking water and other drinking water (DW), the categories are never, 1 to 3 days per week, 4 to 6 days per week, and every day.

Fig. 3 shows the frequency of self-reported behavior that led to contact with the environment for all the study cities by pathway and age group. Despite clear variation in contact frequencies between cities for all pathways, adults and children in the same city had similar contact frequencies. In Accra, Kumasi, and Kampala, more than 85% of adults and more than 60% of children reported that they bathed more than 10 times per week. More than half of the population reported that they consumed municipal drinking water every day in all the cities except Accra, Siem Reap, and Atlanta. In Siem Reap, all the respondents reported that they mainly relied on other drinking water sources (bottled water or well water) and did not drink municipal water. In Maputo and Siem Reap, only a small proportion of population (<10%) reported that they never had contact with flood water in any given week during the rainy season, but large proportions of population (>75%) reported never having contact with open drains in any given month. In Accra, Dhaka, Kampala, Kumasi, Vellore, and Dakar, the proportion of population that reported that they never came in contact with flood water was similar to the proportion of population that reported that they never had contact with open drains within the same city. Overall, use of public or shared latrines was reported more frequently by children compared to adults in the same city. Consumption of raw produce and street food was common across cities. In Ghana, people in Kumasi reported consuming raw produce and street food more frequently compared to people in Accra. Contact with surface water was rare in most cities. In Dhaka and Siem Reap, more than 50% of respondents reported at least one contact with surface water in a month.

### 3.3 Exposure and dominant pathways

**Fig. 4:**
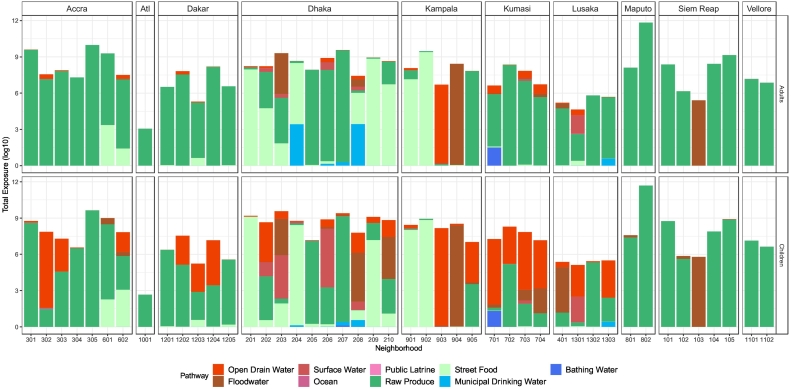
Total fecal exposure by pathway across cities. The height of a bar represents the log scale total exposure from all the pathways while different colors represent the contributions from specific pathways. The unit of exposure to *E. coli* is CFU/MPN per month. The names of neighborhoods that correspond to the neighborhood IDs are shown in Table 4.

Fig. 4 shows average monthly total exposure to fecal contamination and the contribution of each pathway by neighborhood and age group across ten cities in the current study. The magnitude of average total exposure varied within and between cities. Based on reported behavior and measured *E. coli* concentrations, our analyses indicate that most people (both adults and children) in the study cities (except Atlanta and Lusaka) ingested on average more than 10^6^ *E. coli* CFU or MPN per month. For similar levels of total exposure to fecal contamination, the contributions by specific pathways varied by city. For example, in Vellore, only one pathway (i.e., raw produce) contributed the majority of the total fecal exposure. In other cities, multiple pathways made large contributions to the total fecal exposure, such as open drains and produce in Kumasi.

Fig. 4 shows the dominant pathways of fecal exposure, which are defined as those that make substantial contributions (usually >10%) to the total exposure, in the 45 study neighborhoods in the ten cities of this study. Table 4 shows the single most dominant pathway for adults and children by study neighborhood. Produce and street food were very common dominant pathways for exposure to fecal contamination across cities, while open drains and flood water were more site-specific dominant pathways in many neighborhoods. Municipal water, public latrines, and surface water were also identified as dominant pathways in a few study neighborhoods. Some cities such as Accra, Siem Reap, and Vellore had similar dominant pathway(s) across all the study neighborhoods in the city, while in other cities, different neighborhoods had different dominant pathways. For instance, in Kampala, the dominant pathways, including raw produce, street food, open drains, and flood water, were very different between study neighborhoods.

**Table 4:** Dominant pathways^a^ for exposure to fecal contamination identified by neighborhood and age group.

City	Neighborhood	ID	Adult	Children
Atlanta	Peoplestown	1001	Raw produce	Raw produce
Accra	Shiabu	301	Raw produce	Raw produce
	Chorkor	302	Raw produce	Raw produce
	Kokomlemle	303	Raw produce	Raw produce
	Ringway	304	Raw produce	Raw produce
	Adabraka	305	Raw produce	Raw produce
	Mataheko	601	Raw produce	Raw produce
	Osu Alata	602	Raw produce	Raw produce
Dakar	Wakhinane Nimzatt	1201	Raw produce	Raw produce
	Medina Gounass	1202	Raw produce	Raw produce
	Djiddha Thiaroyye Kao	1203	Raw produce	Open drain water
	Rufisque Est	1204	Raw produce	Open drain water
	Sicap Liberte	1205	Raw produce	Raw produce
Dhaka	Kalshi	201	Street food	Street food
	Badda	202	Raw produce	Surface water
	Gabtoli	203	Raw produce	Street food
	Uttarkhan	204	Municipal drinking water	Street food
	Gulshan	205	Raw produce	Raw produce
	Kamalapur	206	Raw produce	Raw produce
	Shampur	207	Raw produce	Raw produce
	Hazaribagh	208	Municipal drinking water	Floodwater
	Motijhil	209	Street food	Street food
	Dhanmondi	210	Raw produce	Street food
Kampala	Makindye	901	Street food	Street food
	Central	902	Street food	Street food
	Kawempe	903	Open drain water	Open drain water
	Rubaga	904	Floodwater	Floodwater
	Nakawa	905	Raw produce	Raw produce
Kumasi	Fante New Town	701	Raw produce	Bathing water
	Moshie Zongo	702	Raw produce	Raw produce
	Dakodwom	703	Raw produce	Open drain water
	Ahodwo	704	Raw produce	Open drain water
Lusaka	Kanyama	401	Raw produce	Floodwater
	Chawama	1301	Surface water	Surface water
	Chazanga	1302	Raw produce	Raw produce
	George	1303	Raw produce	Open drain water
Maputo	Intervention	801	Raw produce	Raw produce
	Control	802	Raw produce	Raw produce
Siem Reap	Chong Kaosou	101	Raw produce	Raw produce
	Kumruthemey (informal)	102	Raw produce	Raw produce
	Kumruthemey (formal)	103	Floodwater	Floodwater
	Steung Thumey	104	Raw produce	Raw produce
	Veal/Trapangses	105	Raw produce	Raw produce
Vellore	Old Town	1101	Raw produce	Raw produce
	Chinna Allapuram	1102	Raw produce	Raw produce

a In neighborhoods with more than one dominant pathway, only the pathway with the greatest contribution to risk is shown in the table.

## 4 Discussion

In many LLMICs, inadequate sanitation and poor fecal sludge management in urban areas lead to widespread fecal contamination in the residential environment. Tools such as the Excreta Flow Diagram (Peal et al., 2020) have been helpful to estimate how much feces is safely managed and how much feces leaks into the environment. However, in many cities, the load of fecal contamination in different compartments (environmental reservoirs), and the ways that people come into contact with those environmental compartments, have not been sufficiently characterized. The current study describes fecal contamination levels in different environmental compartments, frequency of behaviors that lead to exposure to fecal contamination in the environment, and fecal exposure assessments for these pathways across ten cities. The key findings of this study include:

1. Fecal contamination levels in environmental compartments vary across cities.
2. Human behavior that leads to exposure to fecal contamination in the environment differs between cities, but reported behaviors of adults and children are similar within a given city.
3. Food (raw produce and street food) is a universally dominant pathway for exposure to fecal contamination.
4. Open drain water, flood water, and municipal drinking water, are site-specific dominant pathways for exposure to fecal contamination that vary by city and by neighborhood within cities.

### 4.1 Universal dominant pathways

Ingestion of contaminated food is one of the most critical fecal-oral transmission pathways (World Health Organization, 2015; Berger et al., 2010; Rane, 2011), and the results of this study suggest that it could be a universally dominant fecal exposure pathway in urban settings. We define a universal dominant fecal exposure pathway as a pathway that consistently contributes a large proportion of fecal contamination ingestion to the total exposure regardless of geographical location. Essentially, the contribution of food-related pathways to total exposure is considerable across LLMIC cities. Results from the behavioral surveys indicate that the majority of respondents across study cities regularly consume raw produce and street food, and the results from the food samples we analyzed indicated that high fecal contamination levels were common in many cities – even in middle- and higher-income neighborhoods. Given frequent consumption and large amount of ingestion, food, once contaminated, could easily lead to ingestion of fecal contamination and pathogens and may subsequently result in enteric infection or illness. Countries with well-developed surveillance systems for foodborne disease have identified raw produce as a leading cause of foodborne illness (Johnson, 2019; Carstens et al., 2019), and it is likely that this is also true for many LLMIC.

Raw produce and street food can become contaminated at multiple points along the “farm-to-fork” continuum, including application of wastewater irrigation at the farm, and handling and transportation of produce to and within the marketplace (Berger et al., 2010; Drechsel and Keraita, 2014; Ailes et al., 2008). The context-specific nature of food handling, preparation (cooked/uncooked), and consumption practices, along with complex supply chains for raw produce and street food ingredients, makes the reduction of exposure to fecal contamination through food very challenging. As long as a person ingests one serving of highly contaminated raw produce or street food within a month, the monthly exposure from food will have quite a large contribution to the total exposure and easily become a dominant pathway. Even when these food compartments are not identified as the most dominant pathways in a neighborhood or city, raw produce and street food consistently contribute to relatively high levels of fecal exposure. When the exposure from other dominant pathways decreases, raw produce and street food are likely to become dominant pathways.

Although raw produce and street food are universally dominant pathways, approaches to effectively reduce exposure to fecal contamination through these pathways may require site-specific interventions. Critical control points (Food and Agriculture Organization/World Health Organization, 2014a; Food and Agriculture Organization/World Health Organization, 2014b) for food safety hazards from farm to fork may vary by city and even by neighborhood. A study in Accra, Ghana determined that the key factors that impacted produce contamination included the use of wastewater for irrigation, soil contamination at farms, temperature and duration of storage at markets and during transport, and the use of unclean water by market vendors (Antwi-Agyei et al., 2015). A 2014 study by Khairuzzaman et al. (2014) identified key control measures for street food safety in Bangladesh as the provision of safe water and sanitation infrastructure, micro-credits for street food vendor participation in food safety awareness campaigns, and implementation of regulations for vendors who prepare food at home to then sell on the street (Khairuzzaman et al., 2014). Such examples illustrate the importance of site-specific, in-depth exposure assessment along the food pathway at multiple points and demand for further information to determine the critical control points to reduce food contamination. Our study findings illustrate the importance of considering food safety in WASH interventions and environmental health programming.

### 4.2 Site-specific dominant pathways

Many exposure pathways are heavily affected by sanitation infrastructure and human behavior, which differ between and within cities. The open drain water and flood water samples analyzed in this study were highly contaminated across all the study cities. People are more likely to come in contact with open drains and flood water in urban areas with poorly-constructed sewerage systems or no sewerage. These neighborhoods tended to have open drains (e.g., in Accra, Kumasi, Kampala, and Dhaka) and flood water (e.g., in Dhaka, Lusaka, Maputo, and Siem Reap) as dominant pathways of exposure to fecal contamination. The contribution of open drains and flood water may also be seasonal as heavy rainfall in the rainy season increases the chance of contact with open drain water and flood water.

Municipal drinking water also had a pattern of frequent consumption and large volume of ingestion in most of our study cities, but it was not often identified as a dominant pathway due to low fecal contamination in municipal water. Only Dhaka had relatively high fecal contamination levels in the municipal drinking water, possibly due to frequent pipe breaks, illegal connections, and low or negative pressure due to intermittent service (Sirajul Islam et al., 2007; Islam et al., 2010). At times, other drinking water sources serve as the primary drinking water source due to lack of trust in municipal sources, intermittent supply, or long travel distance from dwellings. For example, in Siem Reap, although the contamination level of the municipal drinking water was low, people chose bottled water and well water, which had higher levels of contamination according to our laboratory analyses, as their primary drinking water sources.

### 4.3 Fecal indicator vs. fecal pathogen

These exposure assessments used *E. coli* as a fecal indicator to estimate the amount of fecal contamination ingested through each pathway per month. Some quantitative microbial risk assessment (QMRA) studies (Owusu-Ansah et al., 2017; Labite et al., 2010; World Health Organization, 2006) have attempted to estimate dose of fecal pathogens by using a ratio of fecal indicators (measured) to fecal pathogen (approximated). Although a high fecal indicator concentration in the environment is likely to indicate the presence of fecal pathogens (Amin et al., 2020), the ingestion of a fecal indicator is not necessarily proportional to the ingestion of any fecal pathogen (Korajkic et al., 2018), and the calculated risk estimator (e.g., Disability-Adjusted Life Year) from such studies maybe not accurate. Based on the media in transmission pathways and environmental persistence of fecal pathogens in those media, exposure to fecal pathogens may be more likely/unlikely in one pathway compared to another. Direct measurements of fecal pathogens in the environment are needed to generate appropriate estimates of exposure to fecal pathogens that can be input into dose-response models to predict health risks.

Exposure to fecal contamination as a useful end point has been proposed by several recent studies (Wang et al., 2017; Ngure et al., 2013; Mattioli et al., 2015) for different purposes. The goal of the SaniPath Exposure Assessment Tool is to provide data to support evidence-based decision making about interventions and investments. Instead of providing information on enteric disease burden which may only be the “tip of the iceberg”, this exposure assessment evaluates human exposure to fecal contamination, which is the necessary pre-requisite for enteric infection and illness. Although a concrete relationship between exposure to fecal contamination and developing enteric illness is challenging to establish, ingesting more fecal contamination is likely to indicate a higher chance of ingesting more fecal pathogens and consequently a greater probability of developing enteric infection, illness, and subsequent adverse health outcomes. Although lacking a benchmark of risk (i.e., threshold of exposure to fecal contamination indicator to determine risk), a fecal exposure metric can still be used to evaluate the importance of various pathways and possibly the impact of interventions.

### 4.4 Exposure assessment can guide decisions

The urban environment is complex, dynamic, and varied. Building the evidence base for a diversity of urban settings will help inform sanitation decision making. Unsafely managed fecal contamination is unevenly distributed across different environmental compartments, and this distribution varies by city. Preventing human contact with highly contaminated environmental compartments, such as open drains and flood water, along with reducing contamination in food and drinking water could reduce fecal exposure substantially from those pathways. Our study showed that there was variation in the dominant pathways of exposure across different cities. This reinforces the need for context-specific interventions. Even for the same dominant pathway in two cities, the most strategic intervention may vary based on whether exposure from a particular pathway is driven more by environmental contamination or behavior in a particular context. Efforts to reduce fecal exposure through different pathways could be a mix of long-term government infrastructure investment and improved services and short-term behavior change. In many situations, the service authority's access to resources or incentives may be the fundamental determinant of the “best” intervention approach for a specific pathway.

The results of the SaniPath Tool have provided valuable information to influence and inform local and national policy, and it is important to continue to collect such data to inform targeted decision making at the local and national levels. In Ghana, results from the SaniPath study have been shared widely with WASH sector stakeholders and have impacted policy decision-making and the approach to WASH development, particularly through the inclusion of food safety as part of the National Liquid Waste Management Plan and Kumasi Metropolitan Assembly Annual Sanitation Plan for 2020 (Resource Centre Network Ghana, 2013).

Standardizing and aggregating data across multiple cities, as demonstrated in this study, and the identification of universal exposure pathways, can more broadly inform WASH sector priorities for multilateral organizations, development banks, and investors. For example, a recent World Health Organization (WHO) and UNICEF position paper on WASH and nutrition highlighted the role of contaminated food in the exposure of communities to fecal contamination as a growing concern and cited the impact of SaniPath findings (World Health Organization U., 2019). The paper notes that programs and policies aimed at reducing exposure to fecal contamination should consider the importance of food safety and the safe use of wastewater for irrigation. Bringing attention to otherwise unrecognized or under-estimated pathways of exposure can influence action. Additionally, foundations and development banks may be able to use these insights, along with other evidence, to advance future research and operational priorities. As additional information on exposure to fecal contamination in urban settings is collected, and the relative contribution of different pathways to total exposure is characterized, sanitation investments can be better prioritized to maximize public health benefits.

### 4.5 Strengths and limitations

This article describes the results of assessments of exposure to fecal contamination using a standardized method for ten cities. Thousands of samples from different environmental compartments and behavior surveys from households, communities, and schools were collected. This is a novel and systematic approach to collect behavior information (for both adults and children), paired with environmental compartments, by pathway at a large scale across multiple cities. Such a multi-level framework allows us to compare the results from different angles, by pathway, by age group, by neighborhood, and by city, using a common, standardized exposure metric (estimated *E. coli* CFU/MPN ingested per month) as the outcome. This standardized metric enables comparison of the exposure risk from multiple pathways across multiple sites.

There are several limitations of the current study. First, the SaniPath Tool assessment is a cross-sectional assessment and does not examine the seasonality and temporal variation in exposure to fecal contamination. The deployments presented in this study deliberately targeted the peak diarrhea season or rainy season, and behavior with high seasonality (e.g., frequency of flood water contact) was asked specifically for the rainy season. Second, this study measured *E. coli* as the fecal indicator. In some settings, *E. coli* has adapted to live outside the host. Its proliferation and die off in some environments can complicate the quantitative relationship between fecal contamination and *E. coli* (Liu et al., 2012; Jang et al., 2017). Furthermore, *E. coli* is excreted in both human and animal feces, and the relative contribution of these multiple sources to the fecal contamination in the environment is unclear. While exposure to both human and animal fecal contamination poses a risk to human health, interventions aimed only at human fecal contamination may not remove the greatest source of fecal contamination in some settings. Third, only low-income neighborhoods were selected in some of the study cities, and the results from those areas may not be representative of fecal exposure for the whole city. Finally, while definitions of SaniPath environmental pathways are provided in the Tool guidance material, some pathways vary minimally across contexts, and others can vary quite a bit. For example, flood water is defined in the tool as “water standing for at least 1 hour”. In some contexts, this may be characterized by puddles, but in other more flood-prone areas, this may be characterized by knee-deep water.

### 4.6 Conclusion

Standardized multi-pathway exposure assessments for fecal contamination were conducted from 2014 to 2019 in nine cities in low-income and lower-middle-income countries and one city in the United States. Both environmental samples and behavior surveys were collected and used to estimate exposure to fecal contamination in the environment through different pathways for each study site. Overall, the food (raw produce and street food) pathways were dominant pathways across all study cities, while open drain, flood water, and municipal drinking water made substantial contributions to fecal exposure in certain neighborhoods within individual cities. By utilizing the results from SaniPath Exposure Assessments conducted across countries and regions of the world, an understanding of the impact of poor fecal sludge management on urban residents in LLMIC can be developed and leveraged to inform city-, country-, and region-specific investments and interventions by local and national governments, NGOs, and development banks.

## CRediT authorship contribution statement

**Yuke Wang:** Conceptualization, Methodology, Software, Validation, Formal analysis, Writing – original draft, Writing – review & editing, Visualization, Supervision. **Wolfgang Mairinger:** Methodology, Software, Formal analysis, Data curation, Writing – review & editing, Visualization. **Suraja J. Raj:** Conceptualization, Investigation, Writing – original draft, Writing – review & editing, Supervision, Project administration. **Habib Yakubu:** Conceptualization, Investigation, Writing – review & editing, Supervision, Project administration. **Casey Siesel:** Methodology, Software, Investigation, Writing – original draft, Writing – review & editing, Visualization, Project administration. **Jamie Green:** Investigation, Writing – review & editing, Supervision, Project administration. **Sarah Durry:** Investigation, Writing – review & editing, Supervision, Project administration. **George Joseph:** Investigation, Writing – review & editing, Supervision. **Mahbubur Rahman:** Investigation, Writing – review & editing, Supervision. **Nuhu Amin:** Investigation, Writing – review & editing, Supervision. **Md. Zahidul Hassan:** Investigation, Writing - Review & Editing. **James Wicken:** Investigation, Supervision. **Dany Dourng:** Investigation, Writing – review & editing. **Eugene Larbi:** Investigation, Writing – review & editing, Supervision. **Lady Asantewa B. Adomako:** Investigation, Writing – review & editing. **Ato Kwamena Senayah:** Investigation, Writing – review & editing. **Benjamin Doe:** Investigation, Writing – review & editing, Supervision. **Richard Buamah:** Investigation, Writing – review & editing, Supervision. **Joshua Nii Noye Tetteh-Nortey:** Investigation, Writing – review & editing. **Gagandeep Kang:** Investigation, Supervision. **Arun Karthikeyan:** Investigation, Writing – review & editing. **Sheela Roy:** Investigation, Writing – review & editing. **Joe Brown:** Investigation, Writing – review & editing, Supervision. **Bacelar Muneme:** Investigation, Writing – review & editing. **Seydina O. Sene:** Investigation, Writing – review & editing. **Benedict Tuffuor:** Investigation, Writing – review & editing. **Richard K. Mugambe:** Investigation, Writing – review & editing, Supervision. **Najib Lukooya Bateganya:** Investigation, Writing – review & editing. **Trevor Surridge:** Investigation, Writing – review & editing. **Grace Mwanza Ndashe:** Investigation, Writing – review & editing. **Kunda Ndashe:** Investigation, Writing – review & editing. **Radu Ban:** Conceptualization, Supervision. **Alyse Schrecongost:** Conceptualization, Writing – review & editing. **Christine L. Moe:** Conceptualization, Methodology, Writing – review & editing, Funding acquisition.

## Declaration of competing interest

The authors declare that they have no known competing financial interests or personal relationships that could have appeared to influence the work reported in this paper.

## Appendix A Supplementary data

Supplementary material Image 1
