# Supplementary material for: Quantitative assessment of exposure to fecal contamination in urban environment across nine cities in low-income and lower-middle-income countries and a city in the United States
Source: Sci Total Environ. 2022 Feb 1;806:151273. doi: 10.1016/j.scitotenv.2021.151273 (PMC8651627; doi:10.1016/j.scitotenv.2021.151273)
Supplement: Supplementary file 1 — Supplementary material [file mmc1.docx]

**Supplementary Material**

**S1. Sample Size of Environmental Samples**

Table S1. Numbers of environmental samples collected by sample type and city.

| **City** | **Lab Method** | **Drain Water** | **Flood Water** | **Ocean Water** | **Surface Water** | **Bathing Water** | **Other Drinking Water** | **Municipal Drinking Water** | **Raw Produce** | **Street Food** | **Public Latrine** | **Soil** |
| --- | --- | --- | --- | --- | --- | --- | --- | --- | --- | --- | --- | --- |
| Accra | MF | 184 | 18 | 40 | N/A | 9 | N/A | 88 | 90 | 20 | 280 | 108 |
| Atlanta | IDEXX | N/A | 7 | N/A | N/A | N/A | N/A | 10 | 10 | N/A | 10 | 10 |
| Dakar | MF | 50 | N/A | N/A | N/A | N/A | N/A | 100 | 50 | 50 | N/A | 50 |
| Dhaka | IDEXX | 100 | 100 | N/A | 100 | 100 | 100 | 100 | 100 | 100 | 100 | 100 |
| Kampala | MF | 47 | 50 | N/A | 12 | N/A | 39 | 39 | 50 | 45 | 50 | 50 |
| Kumasi | MF | 40 | 36 | N/A | 3 | 21 | N/A | 36 | 39 | 40 | 27 | 40 |
| Lusaka | IDEXX | 50 | 30 | N/A | 10 | N/A | 90 | 40 | 50 | 50 | 50 | 50 |
| Maputo | MF | 22 | 20 | N/A | N/A | 50 | N/A | 40 | 23 | N/A | 71 | 150 |
| Siem Reap | MF | N/A | 50 | N/A | N/A | 10 | 150 | 10 | 33 | N/A | N/A | 50 |
| Vellore | MF | N/A | N/A | N/A | N/A | 20 | N/A | 22 | 20 | N/A | 24 | 20 |

Types of other drinking water varied by city including shallow well water, well water, borehole water, bottled water, spring water, and ice. N/A represents no samples collected since the exposure pathway was not included in the city.

**S2. Private Toilets**

Table S2 summarizes the results from contextual survey questions about private toilets by city. There was major variation across cities about the reported presence of private toilets in households or compounds. Most respondents in different cities reported using toilet if it was available in the household or compound. Surveys from most of the cities indicated that a high proportion of respondents had flush toilets except in Lusaka (32.3%) and Kampala (6.7%). Many of the respondents in Siem Reap (30.4%) reported that the toilets in their household or compound flooded.

Table S2. Number and percent of respondents answered yes for contextual questions by city.

| **Questions** | **Accra** | **Dakar** | **Dhaka** | **Kampala** | **Kumasi** | **Lusaka** | **Maputo** | **Siem Reap** | **Vellore** |
| --- | --- | --- | --- | --- | --- | --- | --- | --- | --- |
| *Total number of household surveys* | 1021 | 500 | 823 | 548 | 400 | 400 | 261 | 410 | 200 |
| *Do you have any toilets in your household/compound?* | 666 (65.2%) | 499 (99.8%) | 241 (29.3%) | 403 (73.5%) | 145 (36.3%) | 124 (31.0%) | 116 (44.4%) | 359 (87.6%) | 111 (55.5%) |
| **Do you use the toilet in your household/compound?* | 630 (94.6%) | 499  (100.0%) | 235  (97.5%) | 399  (99.0%) | 126  (86.9%) | 124  (100.0%) | 116  (100.0%) | 358  (99.7%) | 108  (97.3%) |
| **Do you flush the toilet with water?* | 609 (91.4%) | 430 (86.2%) | 232 (96.3%) | 27 (6.7%) | 91 (62.8%) | 40 (32.3%) | N/A | 302 (84.1%) | 108 (97.3%) |
| **Does the toilet in your household/compound ever flood?* | 6 (0.9%) | 82 (16.4%) | 18 (7.5%) | 17 (4.2%) | 6 (4.1%) | 20 (16.1%) | N/A | 109 (30.4%) | 1 (0.9%) |
| **Do you share the toilet in your household/compound?* | 263  (39.5%) | 286  (57.3%) | N/A | 150  (37.2%) | 46  (31.7%) | 38  (30.6%) | 98  (84.5%) | 53  (14.8%) | N/A |

*Percentages were calculated using the number of households with toilets in the household/compound as the denominator.
